# An Attempt to Relate Oleogel Properties to Wax Ester Chemical Structures

**DOI:** 10.3390/gels8090579

**Published:** 2022-09-12

**Authors:** Henriette Brykczynski, Birgit Hetzer, Eckhard Flöter

**Affiliations:** 1 Chair of Food Process Engineering, Technische Universität Berlin, 10623 Berlin, Germany; 2 Department of Food Technology and Bioprocess Engineering, Max Rubner-Institut, 76131 Karlsruhe, Germany

**Keywords:** oleogels, natural waxes, wax esters, microstructure, viscoelastic behavior, cooling rate

## Abstract

Wax esters are considered to have a dominant contribution in the gelling properties of wax-based oleogels. To understand their gelling behavior, oleogels of seven different wax esters (total carbon number from 30 to 46; c = 10% [*m*/*m*]) in medium-chain triglycerides oil were characterized. Scanning electron microscopy revealed that wax esters crystallize in rhombic platelets with a thickness of 80 to 115 monomolecular layers. Bright field microscopy showed that the regularity and face length of the crystals increased with the total carbon number and molecular symmetry of the respective wax ester. Oscillatory rheology was used to characterize the gel rigidity ($G_{max}^{*}$). Here, wax ester oleogels with smaller total carbon numbers yielded higher $G_{max}^{*}$ values than those of wax esters with higher total carbon numbers. The gel rigidity ($G_{max}^{*}$) inversely correlated with the crystal face length. Smaller and optically less well-defined platelets promoted higher gel rigidities. In the case of the microstructure of a specific oleogel composition being manipulated by a variation in the cooling rates (0.8; 5; 10 K/min), this relationship persisted. The information compiled in this manuscript further elucidates the crystallization behavior of wax esters in oleogels. This contributes to the understanding of the composition–structure–functionality relationship of wax-based oleogels supporting future food applications.

## 1. Introduction

The current research on alternatives for triglyceride-based structuring of semi-solid fat phases covers numerous different structuring agents [1]. Natural waxes are low-molecular weight oleogelators with broad availability at reasonable procurement costs that form oleogels at low concentrations [2]. Consequently, they are promising candidates for future industrial application. Hitherto, natural waxes such as beeswax (BWX), candelilla wax (CLX) and carnauba wax (CRX) are, for example, already applied as coating agents in confectionery. This implies that they are generally recognized as safe (GRAS-status) [3,4] and are widely accepted by consumers. In addition, other natural waxes such as sunflower wax (SFX) and rice bran wax (RBX) show substantial potential as oleogelators [2,5]. Nonetheless, their application in oleogels remains difficult.

**Figure 1:**
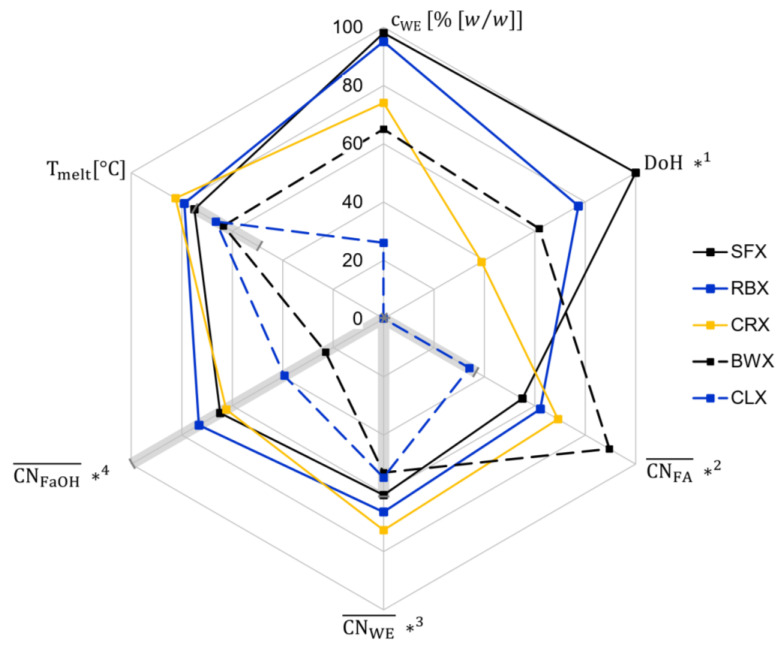
Characteristic parameters of different natural waxes: melting temperature (T_melt_), total wax ester (WE) content (% [*w*/*w*]), degree of homogeneity (DoH), mean total carbon number (CN) of the WE fraction, mean CN of the fatty acid (FA) and fatty alcohol (FaOH) moieties in the WE fraction. Span of axis from center to outer: *1: DoH from 0.23 to 14.27; *2: CN_FA_ from 14 to 24; *3: CN_WE_ from 30 to 56; *4: CN_FaOH_ from 16 to 32. Gray areas: range covered by the WE studied. SFX = sunflower wax, RBX = rice bran wax, CRX = carnauba wax, BWX = beeswax, CLX = candelilla wax.

Natural waxes are composed of hydrocarbons (HC), fatty acids (FA), fatty alcohols (FaOH), wax esters (WE) and some minor components. The proportions of HC, FA, FaOH and WE present in a wax depend not only on the originating plant but also on the extraction and purification processes. Undoubtedly, the oil structuring abilities of waxes result from the overall wax composition. However, long-chained WE are considered dominant for gelling behavior. Figure 1 illustrates characteristic properties of the aforementioned natural waxes. The literature data on the melting temperature [2,6], WE mass fraction [2,7] and characteristics concerning WE fraction are displayed. The data presented on the degree of homogeneity (DoH) [8] and the average total carbon numbers (CN) of FA, WE and FaOH fraction [7] are normalized to ranges typical for natural waxes. The DoH is a parameter defining the homogeneity of a wax, considering the WE concentration as well as the chain length distribution within the WE fraction, as described, in detail, elsewhere [8]. For natural waxes, the DoH ranges between 0.23 (CLX) and 14.27 (SFX). Accordingly, this range is depicted in Figure 1 with CLX located in the center (0%) and SFX at the outer of the scale (100%). The values given for the averaged mean CN of the WE fraction as well as for the FA and FaOH constituting the WE fraction were based on data given by Doan et al. [7]. The axes for these properties were normalized such that the single FA occurring between a CN of 14 and 24 and a single FaOH with a CN between 16 and 32 could be depicted. The mean CN of the WE fraction was calculated assuming a statistical recombination of the respective FA and FaOH moieties, as previously described [9]. In line with the other parameters, the axis for the mean CN of the WE fraction was from the shortest CN present (CN = 30) to the longest CN present (CN = 56). The fat gray shaded areas indicate, respectively, which range is covered by the pure WE studied in this contribution.

The apparent compositional variation of natural waxes propagates into their respective performance as oleogelators. It is well established that wax-based oleogels are particle gels based on the aggregation of wax crystals forming a three-dimensional network [1,10]. The structure and functionality of wax-based oleogels has extensively been studied by several authors, e.g., [5,11,12] but cannot be reviewed within this study.

Irrespective of the type of molecules–WE, FA, FaOH or HC–the crystal structure results from the parallel assembly of alkyl chains [13,14]. The effectiveness in oleogelation is related to the platelet-like crystal habit. The crystal habit is driven by the growth rates in different directions. Dassanayake et al. previously emphasized this during the discussion of synchrotron radiation X-ray spectra and micrographs of oleogels prepared with RBX and salad oil [15].

During gel formation, nano-platelets aggregate to form a three-dimensional network [16]. It has been reported that the particle network can be manipulated by variation of, e.g., the oil to be entrapped [8,17] and the process conditions applied [18,19]. The description of the three-dimensional scaffolding is not univocal. Cardhouse-like and spherulitic structures are mainly considered [2,20,21,22], but also dendritic networks are described [11]. The platelet-based cardhouse networks are often interpreted to be needle-based. This misinterpretation of light–microscopic images was previously pointed out by Hwang et al. in 2015 [23] and repeatedly confirmed, e.g., [9,18,24].

The differences in gelling behavior and microstructure displayed by different natural waxes is related to variations in wax composition [20,25]. Nonetheless, until now, no relationship between the molecular composition, the process parameters and the resulting structure and functionality for wax-based oleogels has been identified. Many recent publications indicate WE to be decisive for the network formation of natural waxes [9,23,26]. In a previous contribution of ours [9], the relevance of WE crystallization for wax-based oleogels was illustrated by comparing the microstructures of either WE-based or SFX-based oleogels. Similar crystal habits could be generated by the adaption of the cooling rate. This illustrates the relevance of the crystallization of pure WE for oleogels based on natural waxes [9].

**Figure 2:**
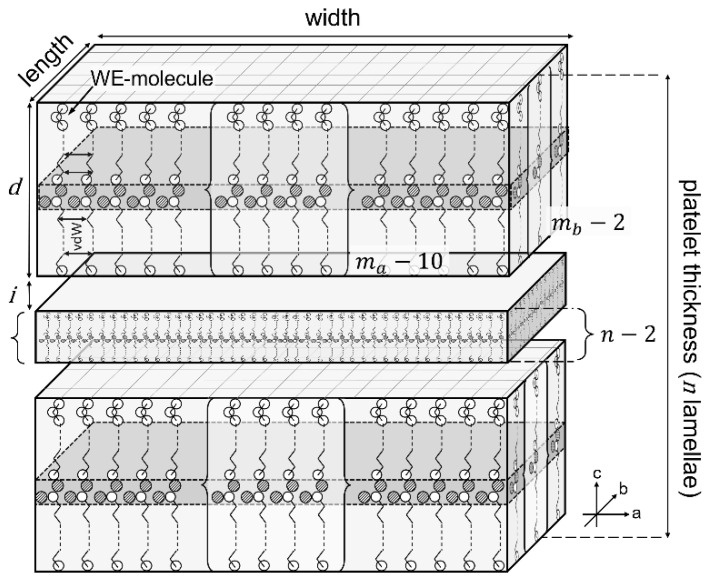
Schematic representation of WE crystal platelets built up of n stacked single lamellae each being composed of $m_{a}\;*\;m_{b}$ individual WE molecules in monomolecular layers. The platelet thickness results from the height of the WE molecules (*d*), the number of lamellae (*n*) and the interlamellar distance i.

A WE crystal is composed of n stacked lamellae, each composed of m individual WE molecules, arranged in monomolecular layers (lamellae). This is schematically illustrated by Figure 2. The crystal surface per mass of solid material essentially results from the planar extension, here simplified as length and width (a and b axis) of the crystal and the platelet thickness (c axis). This depends on the number of stacked lamellae and the lamellar thickness. These theoretical considerations lead to the conclusion that the CN has a profound effect on the crystal size and hence, the crystallization behavior of WE: the higher the CN, the larger the size of a single WE molecule and thus, the lamella thickness d. Simultaneously, the lateral interactions between the alkyl chains, predominantly van der Waals (vdW) interactions (indicated by black arrows), also increase. Assuming an equal mass of solid material and a constant number of lamellae in a crystal, the crystal surface is reduced with the increasing molecular weight of the WE. This is because the number of ester bonds in the system is reduced.

The C design of wax-based oil structuring, particularly the solid phase mixing behavior, has yet to be understood. So far, this has not been studied much for oleogels based on pure WE. In this context, the influence of the chain length distribution and the symmetry of the respective WE molecules needs to be unraveled.

Recently, a first study by Avendaño-Vasquez et al. [27] reported the thermal, viscoelastic and microstructural properties of oleogels based on symmetric (CN 28; 32; 36; 40; 44) and asymmetric (FaOH_FA: 18_14; 18_16; 18_20; 18_22) WE. Previously, it was shown that pure WE form oleogels in high oleic safflower oil at low inclusion levels of 3% [*w/w*] [27]. Even though the publication is fairly comprehensive in its approach, revealing a systematic behavior depending on the WE structure, CN and related X-ray data, many questions remain open. The thermal properties of oleogels based on pure WE given herein and the pure WE given in another contribution [9] correspond sufficiently well. Both contributions also find that the maximum elastic moduli ($G^{\prime }$) of WE-based oleogels decrease with increasing CN.

However, based on earlier X-ray diffraction data on pure WE [28,29], it was summarized that WE crystallize in a monomolecular chain packing, either in an orthogonal or a tilted orientation of the alkyl chains within the layer [9]. The existing data indicate that the preferred polymorph is a function of the position of the ester bond within the WE molecule): the orthogonal chain arrangement seems to occur only for WE with a FaOH moiety two carbon atoms longer (∆CN=+2) or four carbon atoms shorter (∆CN=−4) than the FA moiety [9]. This is actually not in agreement with the data given in the contribution by Avendaño-Vasquez et al. [27]. Anyhow, variations in the (micro-) structure of WE-based oleogels can result not only from asymmetry but also from different polymorphs and crystal habits. This lack of certainty requires more investigations of the viscoelastic behavior and the microstructure of WE-based oleogels.

Consequently, this manuscript focuses on the viscoelastic behavior and the microstructure of WE-based oleogels. The WE investigated cover a range from 30 to 46 carbon atoms. According to pre-existing knowledge [9], all but one WE crystallize in an orthogonal chain arrangement. Hence, the difference between the CN of the FaOH and the FA residue, further designated as ΔCN, is either +2 or −4. This paper intends investigating the effect of CN independently of the respective polymorph. To eliminate the effect of differences in the number of ester bonds present in the system on the viscoelastic behavior, the dosage is on a molar basis. The microstructure of oleogels based on different WE is examined with respect to the size and shape of the crystal structures. Hence, this research aims to find a relationship between the microstructure and the resulting viscoelastic behavior.

## 2. Results and Discussion

### 2.1. Viscoelastic Behavior

In earlier publications, oleogels based on WE of different CNs were prepared on a weight basis [9,27]. Accordingly, oleogels based on WE and MCT oil were investigated at a WE inclusion level of 10% [*w*/*w*] in our previous contribution [9]. For weight fraction-based equal dosage, it remains unclear whether the reduced number of WE molecules with increasing molecular weight of the WE is responsible for the decrease in the maximum complex shear modulus ($G_{max}^{*}$). To eliminate this possible contribution, dosage was chosen to be equimolar in this study. Consequently, the number of ester bonds, potentially defining the total planar crystal area remained constant throughout the experimental plan. In Table 1, the WE weight fractions to the respective 10% molar fraction are given.

**Figure 3:**
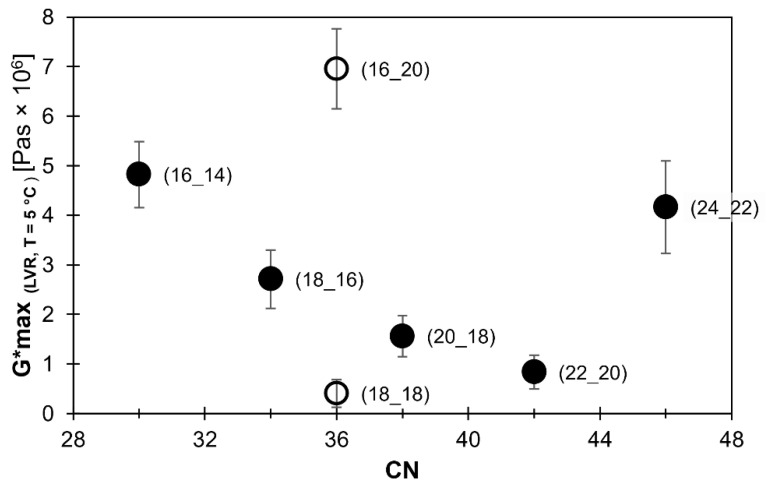
Maximum complex shear modulus ($G_{max}^{*}$) (in linear viscoelastic range (LVR); T = 5 °C) as a function of CN for mono-ester gels consisting of a pure WE (10% [*m*/*m*]) and medium chain triglycerides (MCT) oil. Filled circles: homologous series of ∆CN=+2, hollow circles: not part of homologous series. All WE are denoted as (FaOH_FA).

All oleogel systems were studied within their linear viscoelastic range (LVR). This was ensured using a prior amplitude strain test. (Data not shown.) The maximum complex shear modulus ($G_{max}^{*}$) was determined after 30 min crystallization time at 5 °C. The values which were determined are shown as a function of CN in Figure 3. In addition, with the molar dosage, $G_{max}^{*}$, in general, decreased with increasing CN. This confirms the earlier findings which were based on dosage on an equal weight basis [9,27]. Within the homologous series, ∆CN=+2, the relationship between $G_{max}^{*}$ and CN appeared to be linear in the range from CN 30 to CN 42. $G_{max}^{*}$ decreased from 4.82 × 10^6^ Pas (CN 30 (16_14)) to 8.39 × 10^5^ Pas (CN 42 (22_20)).

DSC measurements of WE-based oleogels confirmed that the WE solubility in MCT oil decreases with increasing CN [9]. Taken that all gels are crystallized at the same temperature, this results in higher supersaturations for higher CNs and hence, relates to higher nucleation rates. It is reasoned elsewhere that the incorporation time into a crystal lattice is longer for larger molecules than for smaller molecules, further promoting smaller crystals for larger WE [30]. Consequently, one would expect smaller crystals for WE with higher CN and hence, $G_{max}^{*}$ to increase with increasing CN. The observations made actually demonstrated the opposite trend. However, it is remarkable that the longest WE studied (CN 46 (24_22)) yielded a considerably higher value, $G_{max}^{*}$ _(CN 46 (24_22))_ = 4.16 × 10^6^ Pas, than the approximately linear correlation covering the other WE of the homologous series suggests. Hence, there were dramatic changes observed on the increase in CN: first, a linear decrease and then, a step change from CN 42 to 46. Thus, the observations made cannot be straightforwardly related to considerations of supersaturation, nucleation and growth rates, as the crystallization theory implies [31,32].

Figure 3 further illustrates the effect of ΔCN. At CN 36, two WE with different ∆CN, thus different lengths of the respective FA or FaOH chain, were investigated. WE CN 36 (18_18), ΔCN=0, yielded the lowest value obtained ($G_{max}^{*}$_(CN 36 (18_18)(18_18))_ = 4.08 × 10^5^ Pas). In contrast, the WE CN 36 (16_20), ΔCN=−4, exhibited the highest $G_{max}^{*}$ value ($G_{max}^{*}$ _(CN 36 (16_20))_) = 6.95 × 10^6^ Pas). Comparison of the data revealed that the $G_{max}^{*}$ values of WE with equal CN but different ΔCN varied within a broader range than the $G_{max}^{*}$ values of the WE of the homologous series in the CN range considered in this study. Accordingly, these data emphasize the importance of molecular symmetry (∆CN) on the structuring performance of WE.

Overall, these observations are in line with earlier oleogel systems studies. Further, this shows that the effect of dosage on either weight or molar basis is insignificant. It was previously reported that molecular symmetry matters. This was indicated by symmetric WE (∆CN=0) showing lower values of viscoelastic parameters than respective asymmetric WE at equal CN [9,27]. For instance, it was found that oleogels with WE at CN 36 are the softest oleogels at ∆CN=0 and most rigid at ∆CN=8 [9].

It is noteworthy to realize that the $G_{max}^{*}$ values found for WE-based oleogels are comparable to those based on natural waxes [8,33] and shortenings based on fully hydrogenated oils [34]. Several parameters contribute to the viscoelastic behavior of oleogels. For TAG-based semi-solid phases, it is widely accepted that van der Waals forces are the predominant attractive interaction between nanocrystalline particles dispersed in the oil phase [35,36]. It is fair to assume that WE crystals gel oils according to the same principles. The solid mass fraction is obviously determined by the WE inclusion level and the solubility of WE [37,38,39,40]. However, the latter relates to the CN, symmetry and polymorph of WE considered. The structure efficiency additionally depends on the crystal habit and crystal dimension-driven contributions.

In summary, the hypothesis that the $G_{max}^{*}$ levels of WE-based oleogels would increase with increasing CN at equimolar dosage (% [*m/m*]) was shown to be wrong. The dosage changes, compared to previous studies, were between minus 1% and plus 3% [*w*/*w*] (Table 1). 

This only marginally affected the gel rigidity, emphasizing once more the importance of the WE molecular symmetry for WE-based oil structuring. Hence, a more in-depth understanding of the crystals and the crystal–crystal interaction is required to understand and control wax-based oleogels.

### 2.2. Microstructure

#### 2.2.1. Cryo-Scanning Electron Microscopy (C-SEM)

**Figure 4:**
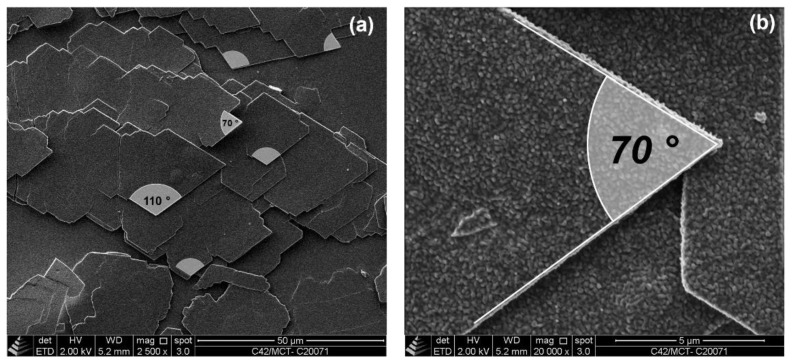
Scanning electron microscopy (SEM) micrographs of WE CN 42 (22_20) for a de-oiled 10% [*w*/*w*] oleogel sample: 2500× magnification (**a**); 20,000× magnification (**b**).

Cryo-SEM images of a de-oiled sample of WE CN 42 (22_20) are shown in Figure 4. The micrographs illustrate that the WE crystals formed in MCT oil are well-defined platelets with straight faces. It appears that the crystal habit is rudimentarily rhombic, but the distinction of single crystals is quite obscured so that a separation of primary crystals is difficult. Individual, very thin plate-like crystals lie on top of each other. The apparent absence of a three-dimensional network structure is possibly an artifact induced by the preparation procedure. Figure 4a (2500× magnification) reveals that the planar area predominantly includes angles between the crystal faces of 70° or 110°. The striking perfection of the crystal faces is illustrated by Figure 4b, showing a well-defined angle of 70°. Beside a rough appearance, caused by the platinum coating, the crystal surface shows no defects, and the faces of the crystal are straight.

This observation on the crystal habit is in line with the description of palmityl palmitate (CN 36 (18_18)) single crystals by Kohlhaas [41]. These were characterized to be thin rhombic platelets with rhombic angles of 74.1° and 106.5°, which is different from the angles observable in Figure 4. The minute differences in the angles found might be due to the crystallization process, effects of the solvent or even the angle of observation.

**Figure 5:**
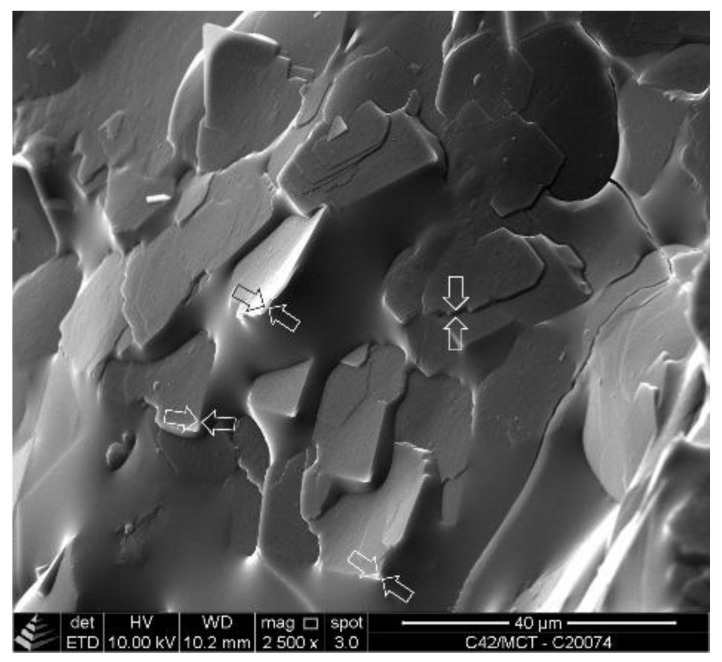
SEM micrograph of oleogel containing MCT oil and 10% [*w*/*w*] of WE CN 42 (22_20) (2500× magnification). Arrows: measuring positions for the determination of the crystal thickness.

Figure 5 shows the cryo-SEM micrograph of an oleogel sample containing MCT oil and 10% [*w*/*w*] of WE CN 42 (22_20) at 2500× magnification. The crystal habit observed above (Figure 4) can also be identified despite the remaining oil droplet. In this partially de-oiled sample, the spatial orientation of the crystals appears fairly random. Neither solid bridges between individual crystals nor a distinct three-dimensional network structure is identifiable.

The thickness of single platelets was determined based on different crystals oriented orthogonally to the projection area (indicated by arrows Figure 5). The values derived are between 460 and 570 nm.

**Figure 6:**
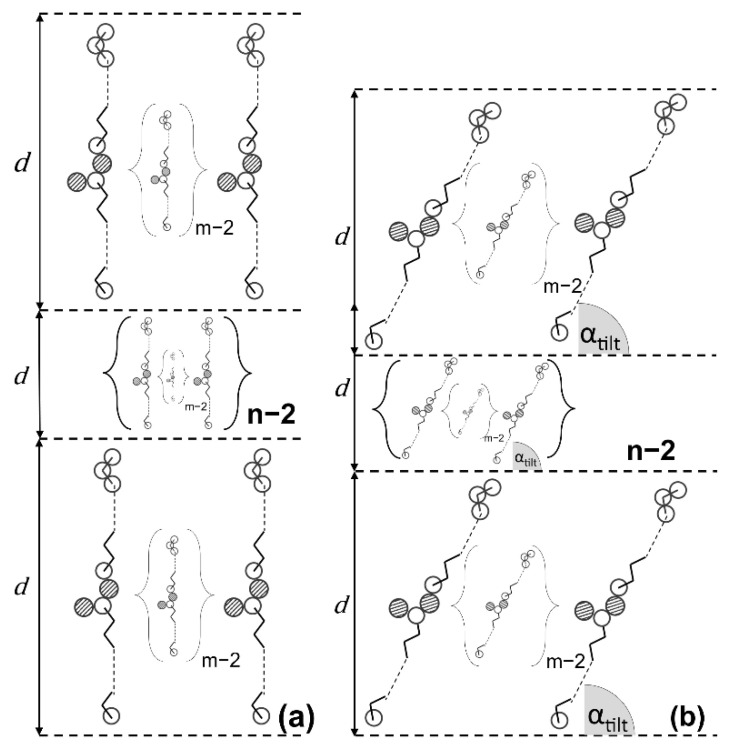
Schematic representation of WE molecules in unimolecular stacking: orthogonal (**a**) or tilted (**b**) chain arrangement related to the methyl end plane. White empty circles: carbon atoms, hatched circles: oxygen atoms, d = layer thickness, m = number of molecules in lateral direction, n = number of molecular layers.

These thickness values and layer thickness values calculated based on earlier publications [9,42] were used to derive the number of molecular layers. This resulted in 80 to 100 molecular WE layers for the orthogonal chain arrangement (Figure 6a) and 90 to 112 molecular WE layers assuming a tilted chain arrangement (Figure 6b).

Both the platelet thickness and number of stacked unimolecular layers are significantly higher than assessed for triglyceride (TAG) crystals [35]. For crystals of fully hydrogenated canola oil, Acevedo et al. [35] stated a stacking of eight lamellae which relates to crystal thicknesses much smaller (factor 15) for the TAGs than derived here for the WE.

Hence, the presented data suggest different lateral and vertical growth rates for the different aliphatic molecules. In a first approximation, possibly ignoring the effects of different growth regimes, it appears unlikely that the methyl end planes of TAGs and WE differ too much [43]. This could indicate similar growth rates orthogonal to the methyl end planes. It is worth noting that TAGs are tri-esters and thus have one ester bond per alkyl chain, whereas WE are mono-esters and have only one ester bond per two alkyl chains.

This gives a first indication that the alkyl chain to ester bond ratio affects growth rates in the planar direction. However, the lamellar stacking of WE crystals certainly requires more detailed consideration.

To discuss this initial finding (i.e., the ratio of alkyl chain to ester bond in aliphatic chain crystals seem to influence the lamellar stacking in the crystalline structure) is certainly beyond the scope of this contribution but warrants further investigations.

#### 2.2.2. Bright Field Microscopy (BFM)

**Figure 7:**
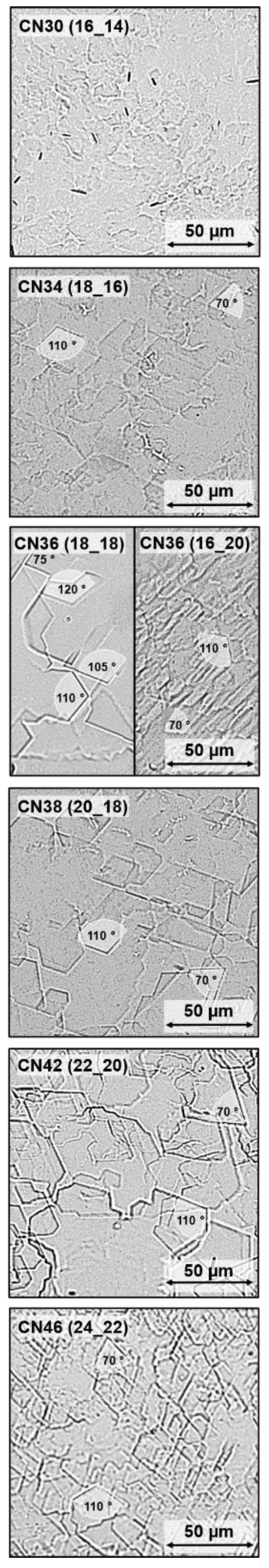
Bright field microscopy (BFM) images of oleogels containing MCT oil and 10% [*m*/*m*] of WE of different CN, indicated in the respective BFM micrograph. Cooling rate: 5 K/min. All WE are denoted as CN (FaOH_FA).

The BFM images of oleogel samples containing MCT oil and 10% [*m*/*m*] of a single WE of different CNs are given in Figure 7. The WE with the lowest CN (CN 30 (16_14)) is on top, and the WE with the highest CN (CN 46 (24_22)) is at the bottom. Herein, the platelet-like morphology visible in the cryo-SEM images is confirmed. Closer examination reveals that crystal size and pronunciation of the well-defined habit increase with CN. Accordingly, the microstructure of WE CN 30 (16_14) has a low regularity, not revealing defined angles or long straight edges. The usually rhombic morphology cannot be identified. Only for this WE, needle-like crystal structures, as described for SFX–oleogels, are discernible. Conversely, all larger WE develop crystals with longer crystal faces and appear as fused rhombic structures. Consistent with the cryo-SEM images, the angles of WE CN 42 (22_20) are found to be 110° and 70°. Most other WE show essentially the same angles. Within the WE with CN 36, remarkably different microstructures are observed. The symmetric WE CN 36 (18_18) exhibits large platelets. Here, in addition to 110° and 70°, other obtuse angles such as 120° and 105° were also found. In contrast, the WE CN 36 (16_20) exhibits shorter crystal faces and a much less regular morphology. The angles identified are again 110° and 70°.

Due to the thickness of the specimen and single platelets being grown together, it is impossible to characterize single crystals. To further quantify the morphology, the length of the crystal faces (L_face_) was determined using ImageJ. For each WE, the respective visible maximum face length was determined as the average of the ten longest faces identifiable in Figure 7. These characteristic values representing the crystal size are listed in Table 2.

**Figure 8:**
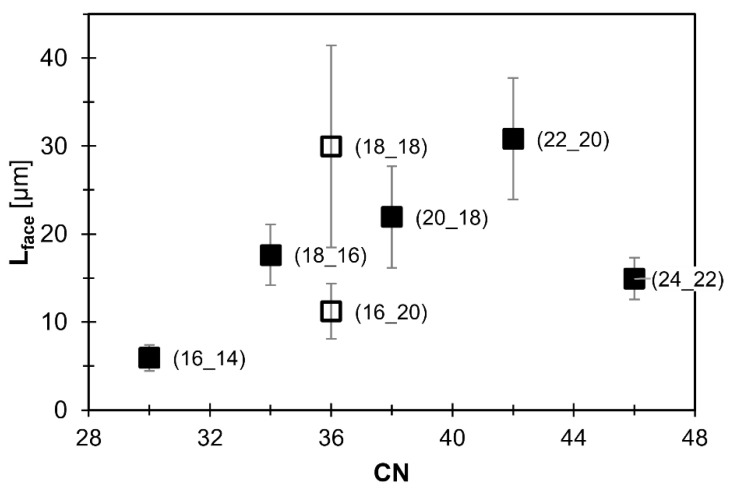
L_face_ of WE crystals in oleogels containing MCT oil and 10% [*m*/*m*] of WE (different CN) as a function of CN. L_face_ was determined using measurement of the ten longest straight faces. The oleogel samples were crystallized at a cooling rate of 5 K/min. Filled squares: homologous series of ∆CN=+2, hollow squares: not part of the homologous series. All WE are denoted as (FaOH_FA).

The relationship of the determined face length and the respective CN is illustrated by Figure 8. The pattern found is qualitatively inverted to that shown and described in Figure 3. Again, the homologous series from WE CN 30 (16_14) to WE CN 42 (22_20) suggests a linear relationship. Along this line, the face length increases by a factor of five. Analogous to Figure 3, the longest WE CN 46 [22_24] deviates from this pattern, showing significantly shorter face lengths than the correlation would suggest. Likewise, the data on the face length emphasize that the symmetry (∆CN) of a WE is of utmost importance. As visible in the micrographs (Figure 7), WE CN 36 (16_20) has much shorter face lengths than CN 36 (18_18).

### 2.3. Relationship between Microstructure and Viscoelastic Behavior

**Figure 9:**
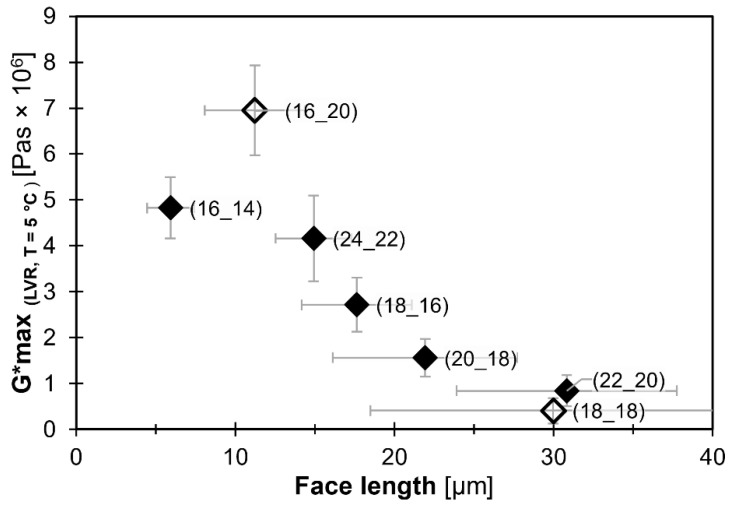
Respective level of $G_{max}^{*}$ (within LVR; T = 5 °C) as function of L_face_ for mono-ester gels (10% [*m*/*m*] WE in MCT oil). Cooling rate: 5 K/min. Filled diamonds: homologous series of ∆CN=+2, hollow diamonds: not part of homologous series. All WE are denoted as (FaOH_FA).

The simplest attempt to correlate characteristic parameters on the microstructure and the viscoelastic behavior is presented in Figure 9. The figure reveals a surprisingly strong relationship. Except for the oleogel based on WE C36 (16_20), the data suggest a linear dependency of the $G_{max}^{*}$ on the face length. Obviously, this simple relationship is rather an artifact from scaling the face length to the internal surface and more precisely, to rheological properties. Anyhow, this finding indicates that the interaction of the crystals in the three-dimensional network structure does not change with the CN of WE. Accordingly, it is reasonable to assume that the variation in structure efficiency for different WE is primarily in their crystal size. The two WE with equal CN but different symmetry, CN 36 (18_18)(18_18) and CN 36 (16_20), are already distinguished by a different microstructural appearance (Figure 7). Anyhow, the data for these respective oleogels adhere to the relationship displayed in Figure 9. This documents the importance of the planar extension of WE platelets for the gelling ability of WE.

### 2.4. Effect of Cooling Rate

For two oleogel systems, containing either 10% [*w*/*w*] WE CN 30 (16_14) or CN 42 (22_20), the effect of crystallizing at different cooling rates was assessed. The viscoelastic behavior ($G_{max}^{*}$) and microstructure (including the maximum face length) of the oleogels were determined.

**Figure 10:**
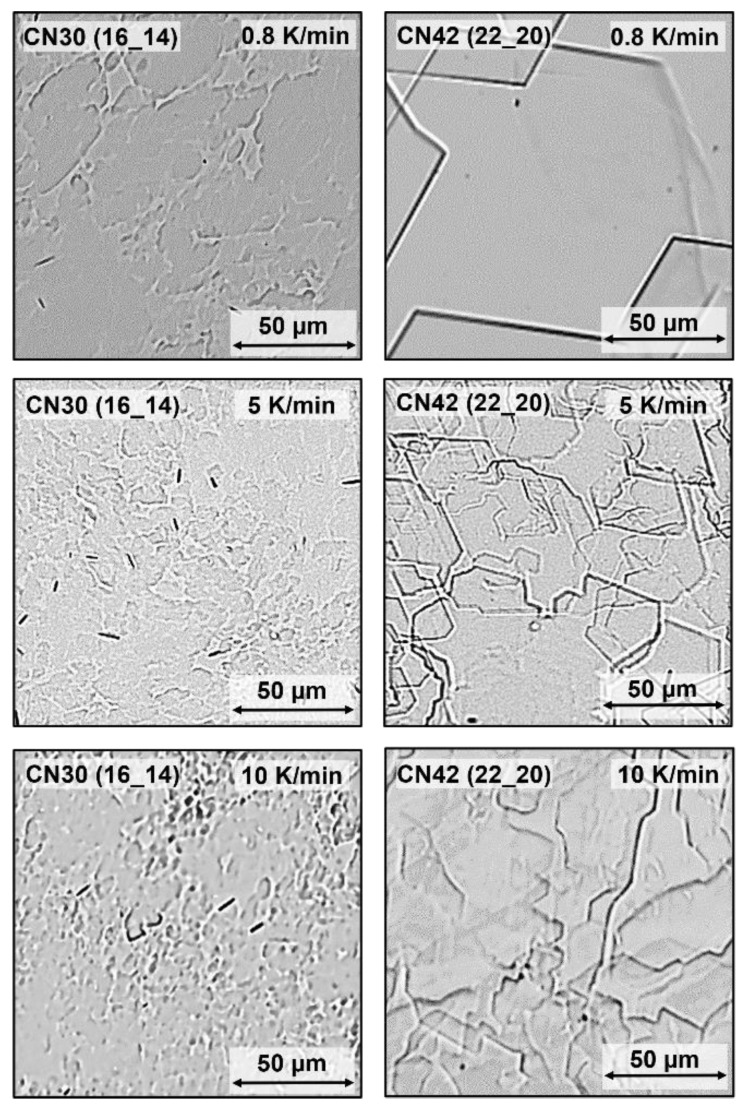
BFM images of WE oleogels (10% [*w*/*w*] of WE CN 30 (16_14) or CN 42 (22_20) and MCT oil) crystallized at three different cooling rates. The WE are denoted as CN (FaOH_FA).

The microstructure of the respective oleogels is illustrated by the BFM images shown in Figure 10. From top to bottom, the cooling rate increases from 0.8 K/min (slow) via 5 K/min (standard rate, Figure 7) to 10 K/min (fast). At any cooling rate, for CN 30 (16_14), neither clear straight edges nor the usual rhombic morphology could be clearly identified. However, for both WE, higher cooling rates (bottom) yield less regular and smaller platelets with shorter faces. This effect is clearly visible for WE CN 42 (22_20) due to better distinguishable structures. These micrographs prove that for WE also, the microstructure does not only change as a function of the molecular make-up but can also be manipulated by variation of the crystallization process. As generally established, increasing cooling rates result in higher nucleation rates and reduced induction times [24]. Furthermore, the results confirm that low cooling rates appear to induce more regular crystal habits as shown elsewhere [9]. The data presented here and in an earlier contribution [9] point out how arbitrary choices of cooling rates and applied supersaturations actually are.

**Figure 11:**
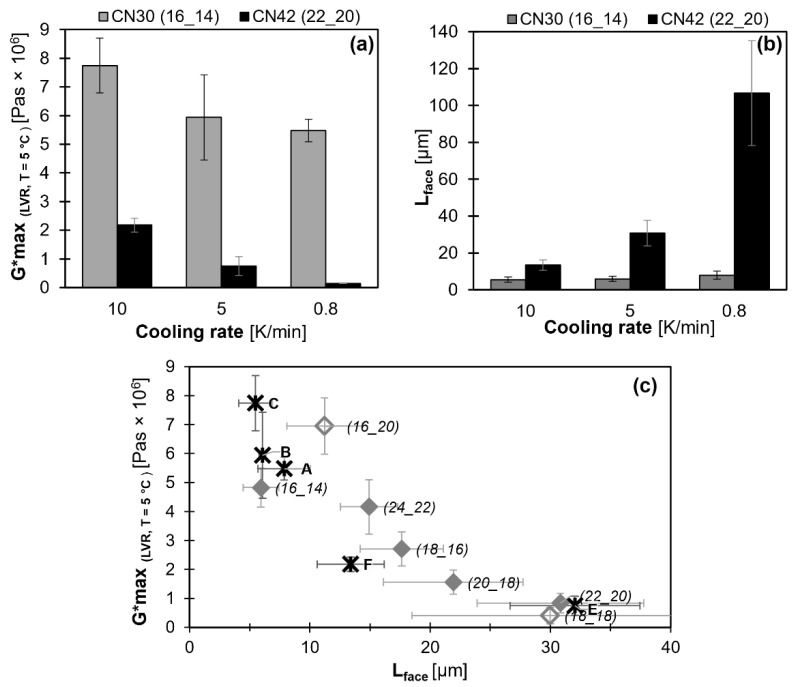
Effect of the cooling rate on mono-ester gels (10% [*w*/*w*] of the respective WE and MCT oil) for (**a**) $G_{max}^{*}$ (within LVR; T = 5 °C) and (**b**) L_face_; (**c**) relationship between microstructure (L_face_) and viscoelastic behavior ($G_{max}^{*}$ ). A: CN 30; 0.8 K/min, B: CN 30; 5 K/min, C: CN 30; 10 K/min, D: CN 42; 0.8 K/min exceeds the range of face length displayed and is not shown, E: CN 42; 5 K/min, F: CN 42; 10 K/min. Filled diamonds: homologous series of ∆CN=+2, hollow diamonds: not part of homologous series. All WE are denoted as (FaOH_FA).

Figure 11a illustrates that the $G_{max}^{*}$ values of the oleogels increase with an increasing cooling rate. The maximum crystal face length in the respective oleogels also systematically changes, as illustrated by Figure 11b. These data are in line with expectations based on generally acknowledged crystallization kinetics, e.g., for wax-based oleogels [24,25]. They confirm that smaller crystals in compositionally equal systems typically yield more rigid gels. Figure 11c is an extension of Figure 9 and illustrates that WE oleogels produced at different cooling rates adhere to the relationship displayed in Figure 9: smaller crystals yield more rigid gels. It emphasizes the validity of the relationship found between face length and $G_{max}^{*}$, irrespective of WE-type and cooling rate.

The data reveal that, in the range studied, the effect of cooling rate is not able to compensate the inferior structuring potential of CN 42 (22_20): even the $G_{max}^{*}$ value of WE CN 30 (16_14) ($G_{max}^{*}$_(CN 30 (16_14), 0.8 K/min)_ = 5.48 × 10^6^ Pas) at the lowest cooling rate is higher than the $G_{max}^{*}$ value of WE CN 42 (22_20) ($G_{max}^{*}$_(CN 42 (22_20), 10 K/min)_ = 2.18 × 10^6^ Pas) at the highest cooling rate. This illustrates that even though the effect of cooling rate is strong, it is not sufficient to compensate the material specific crystallization behavior in the range studied.

This study reveals again how difficult it is to perform correct like-for-like comparisons. However, one must acknowledge that, for practical reasons, experiments at realistic cooling rates derived from anticipated applications seem most justified. For stored products, this could mean low cooling rates. In conclusion, the responses of the WE-based oleogels studied at different cooling rates are in line with published data on wax-based oleogels [24,25,44].

## 3. Conclusions

In this contribution, the viscoelastic behavior and microstructure of different WE-based oleogels are discussed. The oleogels were composed of MCT oil and 10% of a pure WE, respectively. The respective chain length of the WE ranged from 30 to 46 carbon atoms. The WE were mainly selected as a homologous series with a carbon number difference (∆CN) of +2. Irrespective of CN, WE with such a molecular symmetry assume an orthogonal chain arrangement in crystals. Additionally, two WE with CN 36, one symmetric (∆CN=0), one asymmetric (∆CN=−4), were investigated. Earlier publications showed that asymmetry entails the least effects on the caloric properties of WE-based oleogels.

To normalize the WE inclusion regarding the number of ester bonds present, mostly samples with an inclusion level of 10% on a molar basis ([*m*/*m*]) were studied. However, changing from mass to molar dosage only marginally affected the viscoelastic oleogel properties. This proved the hypothesis that structuring functionality is driven by the cumulative planar crystal area wrong.

Bright field microscopy and scanning electron microscopy revealed that WE in oleogels crystallize in very regular, almost two-dimensional platelets with a stacking between 80 and 115 monomolecular layers. Among the primary platelet-like crystals, no sintering was observed. Anyhow, the molecular make-up of the WE regarding CN and ∆CN was found to have a strong effect on crystal habit and size. For increasing CN, more distinct and regular crystals were observed. The projection of planar crystals revealed angles between straight faces of consistently 70° and 110° for most WE. It was found that WE-based oleogels in which no regular, rhombic crystals could be identified by BFM exhibit significantly higher $G_{max}^{*}$ values. The length of the identifiable straight faces in BFM images was defined as a characteristic value for crystal size. It was found that within the homologous series of WE, this length decreased linearly with increasing CN.

To study the effect of the cooling rate on gel structure, oleogels of WE CN 30 (16_14) and CN 42 (22_20), respectively, were investigated. In line with expectations, higher cooling rates yielded smaller crystals and higher $G_{max}^{*}$ values. For the rigidity of the oleogels, it was found that $G_{max}^{*}$ strongly correlated with the characteristic size parameter of the crystals, the maximum face length (L_face_). More rigid gels related to smaller crystals. However, all oleogels studied here, irrespective of the WE employed or the cooling rate applied, adhered to the apparently linear relationship of $G_{max}^{*}$ and the characteristic face length. This indicates that in WE-based oleogels, the crystal size is next to dosage as the dominant factor defining gel strength. Furthermore, these findings suggest that the crystal–crystal interaction defining the three-dimensional network structure remains unchanged throughout the experimental set of gels investigated here.

Admittedly, studies on oleogels based on MCT oil and pure WE appear to be of a rather academic nature compared to product applications of wax-based oleogels. However, the observations of these rather pure systems resemble those made for wax-based oleogels. Even though the fundamental insights are already relevant for future wax-based oleogel applications in foods, further extension into mixtures of wax esters is recommended. This way, a knowledge base can be generated, enabling an a priori design of wax-based structuring systems.

## 4. Materials and Methods

### 4.1. Material

The WE used in this study were purchased from Larodan AB (Solna, Sweden): specifically, palmityl myristate (CN 30 (16_14)), stearyl palmitate (CN 34 (18_16)), myristyl behenate (CN 36 (14_22)), stearyl stearate (CN 36 (18_18)), behenyl myristate (CN 36 (22_14)), arachidyl palmitate (CN 36 (20_16)), arachidyl stearate (CN 38 (20_18)), behenyl arachidate (CN 42 (22_20)) and lignoceryl behenate (CN 46 (24_22)), all with a purity >99%. In this manuscript, the carbon number of the FaOH residue is indicated by the first number, and the carbon number of the FA residue is indicated by the second number.

Medium-chain triglycerides (MCT) oil was obtained from Caesar & Lorentz GmbH (Hilden, Germany). By GC analysis of FA methyl esters according to DGF method C-VI 10a (00), the FA composition of the MCT oil was determined to be 56% caprylic acid (10:0) and 44% capric acid (8:0).

All materials were used without further purification or modification.

### 4.2. Methods

#### 4.2.1. Oleogel Preparation

To form oleogels, 10% [*m*/*m*] of a single pure WE were dissolved in MCT oil. The specific WE were selected to investigate the effects of the CN for WE that accept an orthogonal chain arrangement (∆CN=+2 or −4). Further, at CN 36, a symmetric WE (∆CN=0) was studied. In 1.5 mL screw neck vials, 300 mg (weighing precision: ±0.03 mg) of oleogel were prepared as stock solution. The samples were heated (T = 95 °C) under agitation (250 rpm) to ensure complete dissolution. For the different analytical methods applied, the samples were directly transferred to the respective device configuration while still liquid at high temperature. This was done to avoid changes in the gel composition and network structure.

#### 4.2.2. Viscoelastic Behavior (Rheology)

The viscoelastic behavior of the oleogel systems was studied on a MCR 302 rheometer (Anton Paar GmbH, Graz, Austria). To avoid slippage, a steel plate–plate geometry with sandblasted surface (PP25-S; d = 25 mm) was used. Accurate temperature control was ensured by a top and base Peltier system of the measurement geometry (measuring gap: 0.2 mm).

For sample preparation, the plate was preheated (T = 90 °C) and around 100 mg of the hot stock solution were poured on the center. The samples were sheared ($\dot{\gamma }$ = 10 s^−1^; t = 5 min) for standardization and then stabilized under quiescent conditions (1 min). Gel-sol transitions were observed during the cooling from 90 °C to 5 °C. Afterwards, an isothermal stabilization step was implemented (T = 5 °C, t = 30 min). After that, the isothermal amplitude strain test was performed from 0.005% to 100% at an angular viscosity of 10 rad/s. Prior to repeating the same procedure, samples were heated to 90 °C and standardized. For all measurements, the angular frequency was 10 rad/s, and for temperature and time tests, the applied strain was 0.005%. For the characterization of the viscoelastic behavior, the heating or cooling rates were 5 K/min. For the study on the effect of cooling rate variation, the stated cooling rates were applied. In order to compensate changes in the sample density, the gap width was automatically regulated during the test (F_N_ = 0 N) and reset to 0.2 mm after each heating step. The crystallization was performed in quadruple, and the amplitude sweep was performed in duplicate.

#### 4.2.3. Microstructure

##### Scanning Electron Microscopy (SEM)

Since the available amount of wax ester oleogel was very low, about 50 mg of the samples were placed on a silicon wafer and partially de-oiled with isobutanol which was poured over the sample. Additionally, individual crystalline platelets from the oleogels were isolated using deposition of a few drops of the retained de-oiling solution (isobutanol with traces of washed out MCT oil) on another silicon wafer. Following freezing by liquid nitrogen, the samples were transferred to the cryo chamber (PP2000 T, Quorum Technologies Ltd., Laughton, UK) and pre-cooled at −135 °C. The samples were sublimated (T = –90 °C, t = 15 min) inside the cryo chamber to eliminate eventual ice contamination on the sample’s surface. The samples were coated with platinum in Argon atmosphere (t = 30 s for coating, current: ca. 5–10 mA). This, in order to minimize charging problems. Lastly, samples were loaded to the cryo stage in the SEM chamber (T = −135 °C). For imaging, Quanta 250 FEG field emission scanning electron microscope (FEI, Brno, Czech Republic) under high vacuum (3 × 10^−7^ mbar) was used with an Everhart–Thornley detector (working distance = 5 mm; accelerating voltage = 10 kV). This procedure was kindly performed by the Department of Food Technology and Bioprocess Engineering, Max Rubner-Institut, Karlsruhe, 76131 Germany.

##### Bright Field Microscopy (BFM)

The objective slides for the sample preparation were pre-heated (T = 90 °C), then a 10 µL-droplet of the hot oleogel stock solution was poured on it, and a cover slip was placed. A thermoelectric stage was used to apply different cooling rates in a temperature range from 90 °C to 5 °C. Stabilization of the samples was performed prior to the image recording (T = 5 °C; t = 1 h). All BFM images were captured at ambient temperature.

## Figures and Tables

**Table 1:** Total weight fraction for wax esters of different total carbon numbers (CN) for an inclusion level of 10% on a molar basis (% [*m/m*]) and, respectively, on a weight basis (% [*w*/*w*]).

Total Carbon Number (CN)	% [*m*/*m*]	% [*w*/*w*]
30	10	8.93
34	10	9.93
36	10	10.42
38	10	10.91
42	10	11.86
46	10	12.80

**Table 2:** Face length (L_face_) WE crystals in mono-ester gels (10% [*m*/*m*] of WE in MCT oil). L_face_: average of ten longest straight faces, respectively. Cooling rate: 5 K/min.

WE (FaOH_FA)	L_face_ [µm]	Standard Deviation
CN 30 (16_14)	5.9	*±1.5*
CN 34 (18_16)	17.6	*±3.5*
CN 36 (18_18)	30.0	*±11.5*
CN 36 (16_20)	11.2	*±3.1*
CN 38 (18_20)	21.9	*±5.8*
CN 42 (22_20)	30.8	*±6.9*
CN 46 (24_22)	14.9	*±2.4*

## Data Availability

Not applicable.
